# Serine and Metabolism Regulation: A Novel Mechanism in Antitumor Immunity and Senescence

**DOI:** 10.14336/AD.2020.0314

**Published:** 2020-12-01

**Authors:** Qi Wu, Xinyue Chen, Juanjuan Li, Shengrong Sun

**Affiliations:** ^1^Department of Breast and Thyroid Surgery, Renmin Hospital of Wuhan University, Wuhan, Hubei, China.

**Keywords:** Serine, metabolism, antitumor immunity and senescence

## Abstract

As one of the nonessential amino acids (NEAAs), serine is involved in the anabolism of multiple macromolecular substances by participating in one-carbon unit metabolism. Thus, rapidly proliferating cells such as tumor cells and activated immune cells are highly dependent on serine. Serine supports the proliferation of various immune cells through multiple pathways to enhance the antitumor immune response. Moreover, serine influences aging specificity in an epigenetic and metabolic manner. In this review, we focus on recent advances in the relationship between serine metabolism, antitumor immunity, and senescence. The metabolic regulation of serine seems to be a key point of intervention in antitumor immunity and aging-related disease, providing an opportunity for several novel therapeutics.

## Introduction

Nonessential amino acids (NEAAs) are the amino acids that human cells can synthesize in vivo. NEAAs contribute to producing energy, providing carbon and nitrogen, and defending against oxidation, which are necessary for growth and survival. We know that some definite metabolic signaling pathways appear to enable cells to synthesize NEAAs de novo. However, rapidly proliferating cells, including cancer cells and several immune cells, require an external supply of some amino acids for aberrant growth. For example, glutamine, a kind of NEAA, has been reported to be important for tumor growth by generating energy, producing other amino acids and controlling reactive oxygen species (ROS) [1]. Some cells do not have enough capacity for the de novo synthesis of NEAAs or fail when competing for NEAAs. In these cases, some core pathways that allow cells to synthesize NEAAs are activated to make cells selectively dependent on endogenous supplies. A deeper understanding of this aspect of cell metabolism will reveal novel therapeutic targets for aging-related diseases, including cancer.

Serine is a crucial NEAA for cell growth and survival, participating in the synthesis of the cell membranes, muscle, and nerve sheath. As a precursor of many essential biomacromolecules, such as phospholipids, ATPs, and nucleic acids, serine is reportedly required for rapidly propagating cells [2]. For tumor cells cultured in vivo, exogenous environmental serine consumption is rapid. With the removal of serine from the culture medium, the intracellular serine levels drop sharply, accompanied by slower cell proliferation [3]. Furthermore, the pathways of serine uptake and synthesis are usually upregulated in cancer, which reflects the dependence of tumor growth on serine. Similar to tumor cells, proliferating immune cells in vitro and in vivo are highly dependent on serine [4]. Some key regulators that mediate serine synthesis and metabolism also affect the growth, proliferation, and differentiation of immune cells [5-7]. Thus, both tumor cells and immune cells require an abundant supply of serine, suggesting that immune cells must compete with tumor cells for limited serine resources during the antitumor immune response.

Age is the leading risk factor for numerous chronic diseases, including various types of cancer [8]. Although the mechanisms of aging remain unclear, senescent cells have been regarded as a core contributor to cancer progression and to age-related diseases [9, 10]. Recently, evidence has shown that exogenous serine prevents age-related deficits and alters underlying memory impairment in the brain [11], and serine contributes to a prolonged lifespan [12]. These findings suggest that intracellular serine seems to play an anti-aging role through complex pathways. Here, we focus on serine metabolism and its effects on the development of antitumor immunity and the senescence process. This review offers insight into ways to explore novel therapeutic treatments for serine-dependent cancers and age-related diseases.

**Figure 1.**
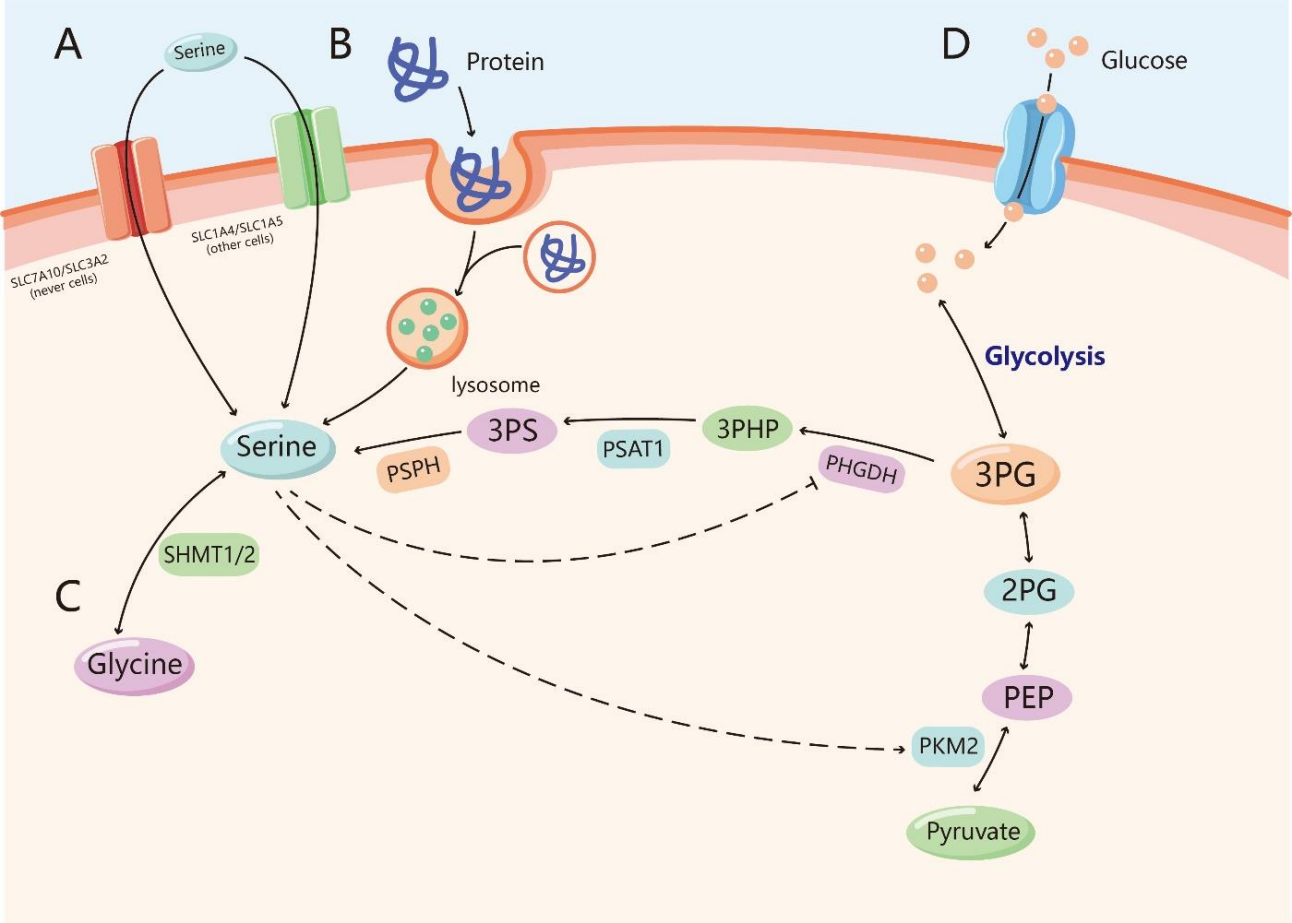
Sources of serine. (A) Serine can be uptaked across cell membranes through the transporters SLC7A10/SLC3A2 (in nerve cells) and SLC1A4/SLC1A5 (in other cells). (B) Serine can be produced by degradation of serine-containing proteins in the lysosome, a process that breaks down proteins which are deactivated or misfolded, such as macropinocytosis and autophagy. (C) Serine can be synthesised from glycine via the catalysis of serine hydroxymethyltransferase (SHMT1/2). (D) Serine can be generated de novo through serine synthesis pathway (SSP). The 3-phosphoglycerate (3-PG) produced by glycolysis or gluconeogenesis is first catalyzed by phosphoglycerate dehydrogenase (PHGDH) to turn into 3-phosphohydroxypyruvate (3-PH), which is combined with a molecule of glutamic acid to produce phosphoserine through the work of phosphoserine aminotransferase 1 (PSAT1), and phosphoserine is finally hydrolyzed into serine by the action of phosphoserine phosphatase (PSPH). Serine directly binds and promotes allosteric activation of the glycolytic enzyme M2 isoform of pyruvate kinase (PKM2), by contrast, PHGDH can be inhibited by serine.

## Sources of serine

Before the function of serine can be understood, knowledge of its source is necessary. Cells acquire serine through four main pathways: uptake from the extracellular environment, degradation from cellular proteins, transamination via glycine, and de novo synthesis from glucose and glutamate. Normally, growing cells require a large amount of serine, which ranks second to the required amount of glutamine among the amino acids [4]. Although extracellular serine is present in the plasma, de novo synthesis of serine can make a fundamental contribution to the supply [13]. Environmental serine deficiency can directly inhibit the proliferation of colorectal cancer cells in vivo and in vitro [3, 14]. Meanwhile, restriction of food-derived serine and glycine has been shown to inhibit tumor growth and improve survival in mouse models of cancer [15]. All of these findings suggest that serine production is very important for cells. However, different tissues and cells depend on different approaches to obtain serine (Fig. 1).

### Serine uptake from the extracellular environment

Serine needs to be taken up across cell membranes through transporters because of its polarity. To date, two pairs of major serine transporters have been identified. One of them is the sodium-independent neutral amino acid exchanger SLC7A10/SLC3A2 [16, 17], which is mainly expressed in the nerve cell membrane. SLC7A10/SLC3A2 has an affinity for both L-serine and D-serine and transports them across the nerve cell membrane [16]. The other pair of serine transporters comprises the sodium-dependent neutral amino acid exchangers SLC1A4/SLC1A5 [18-20], which are expressed outside the nervous system and primarily transport small neutral amino acids, including serine. Recent evidence that cancer cells avidly consume extracellular serine has been obtained in 60 cancer cell lines [4], which indicates that cancer cells need more serine to maintain proliferation and that increasing serine uptake is an important method of serine supplementation. Furthermore, SLC1A4/SLC1A5, the main transporters of serine, are upregulated in serine-dependent tumor cells. The expression of SLC1A4 was reported to be upregulated in breast and lung cancer cell lines [19, 20], while SLC1A5 was overexpressed in multiple tumor tissues, including lung cancer and breast cancer [21, 22]. Moreover, SLC1A5 is crucial for naive T cells, which require rapid amino acid uptake. SLC1A5 deficiency has been shown to impair the differentiation of T helper 1 (Th1) and Th17 cells and attenuate inflammatory responses by inhibiting T cell receptor (TCR)-activated mammalian target of rapamycin complex 1 (mTORC1) [23]. mTORC1 is a nutrient sensor and an important regulator of cell metabolism. It is activated when exogenous growth factors or intracellular nutrients such as serine are abundant [24]. These results suggest that activation and differentiation of T cells are reliable with sufficient amounts of serine. Therefore, the upregulation of serine transporter expression in both tumor cells and T cells is an indispensable process for cellular function.

### Serine obtained from degradation of intracellular and extracellular proteins

Serine can also be produced by the degradation of intracellular and extracellular proteins in the lysosome, a process that breaks down deactivated or misfolded proteins and makes amino acids available for new protein synthesis. For intracellular protein degradation, serine can be obtained from the autophagic degradation of endogenous proteins during acute starvation [25]. However, these processes are more similar to a kind of cellular stress reaction, that occurs when cells are under stress from only the environment or themselves. Cells cannot be self-sufficient and cannot rely solely on the degradation of endogenous proteins to maintain their proliferation. For extracellular protein degradation, pancreatic ductal adenocarcinoma cells with activating KRAS mutations can obtain serine from extracellular proteins that are degraded through macropinocytosis in quantities necessary to meet serine requirements [26]. Likewise, tumor-associated pancreatic stellate cells can provide tumor cells with NEAAs through macroautophagy [27]. Additionally, some cancer cells can scavenge albumin by receptor-mediated macropinocytosis in many breast and prostate cancer cell lines [28]. Albumins are enriched in glutamate and serine, and this scavenging effect replenishes serine in cancer cells, which is beneficial to their anabolism [28]. Based on this phenomenon, we believe that compared with intracellular protein degradation, extracellular protein degradation is a more important process in serine acquisition. We therefore questioned whether tumor cells obtain proteins from other cells or the environment in some manner and then break down exogenous proteins to fuel their supply of serine.

### Glycine transamination and de novo serine synthesis

Serine can be synthesized from glycine via the catalysis of serine hydroxymethyltransferase (SHMT1/2) or generated de novo through the serine synthesis pathway (SSP) from 3-phosphoglycerate (3-PG), an intermediate of glycolysis. Glycine transmission requires a one-carbon unit, but this is not the main approach to acquire serine. In addition to glycolysis, gluconeogenesis-derived 3-PG is the other source of serine from the SSP under fasting conditions [29]. 3-PG produced by glycolysis or gluconeogenesis is first catalyzed by phosphoglycerate dehydrogenase (PHGDH) and converted into 3-phosphohydroxypyruvate (3-PH), which is combined with a molecule of glutamic acid to produce phosphoserine through the activity of phosphoserine aminotransferase 1 (PSAT1), and phosphoserine is finally hydrolyzed into serine by the catalysis of phosphoserine phosphatase (PSPH). The SSP is vital for the acquisition of serine by the brain and tumor cells. Serine is an important neurotransmitter in the brain, yet it is difficult for the brain to acquire serine from plasma due to the structural limitations of the blood-brain barrier [30], so the SSP is the main avenue through which the brain obtains serine. Moreover, tumor cells have high SSP requirements because of their rapid consumption of serine. The SSP is regulated by various factors, including the level of raw materials, serine concentration, and enzyme activity in the synthetic process, which can affect the final rate of serine synthesis [31]. Under serine starvation, cells respond to growth pressure by activating key enzymes to amplify the SSP rate. For example, serine directly binds and promotes allosteric activation of the glycolytic enzyme M2 isoform of pyruvate kinase (PKM2) to augment glycolysis and serine synthesis. PKM2 exists in two forms, and the tetrameric form of PKM2 exhibits high activity, while the monomeric form of PKM2 exhibits low activity [32]. Under serine starvation, PKM2 is transformed into a monomeric state with low activity and slows the last step of glycolysis, leading to the accumulation of upstream glycolysis intermediates such as 3-PG. The accumulation of raw material 3-PG increases the synthesis of intracellular serine [33-35]. In contrast to PKM2, PHGDH can be inhibited by serine [36]. Serine synthesis is accelerated by alleviating the inhibition of PHGDH under serine starvation. Meanwhile, some breast cancer and pancreatic cancer cells overexpress several key enzymes involved in SSP (PSAT1, PSPH, and SHMT2), which leads to increased intracellular serine concentrations, thereby contributing to the long-term maintenance of tumor cell growth [37, 38].

**Figure 2.**
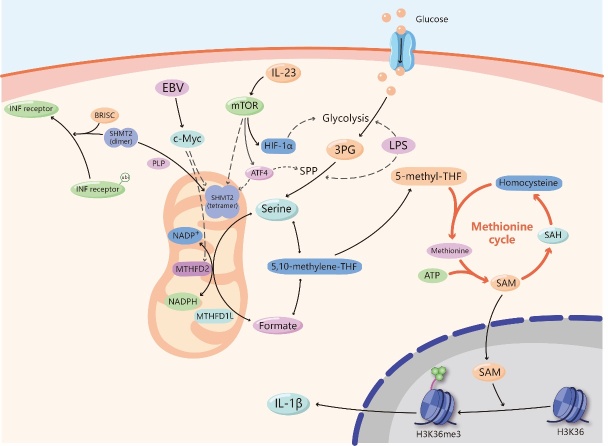
Serine and antitumor immunity. The serine synthesized from 3-phosphoglycerate (3-PG) enters the mitochondrial one-carbon metabolism, which is catalyzed by serine hydroxymethyltransferase 2 (SHMT2), methylenetetrahydrofolate dehydrogenase 2 (MTHFD2) and MTHFD1-like (MTHFD1L) to generate formate. Nicotinamide adenine dinucleotide phosphate (NADPH) is also produced in this process. Formate enters the cytoplasm and transform into 5,10-methylene-THF, then generates 5-methyl-THF. It takes part in methionine cycle, passes the methyl to homocysteine and produces methionine, which generates S-adenosylmethionine (SAM) finally under the activation of ATP. SAM enters the nucleus and is involved in the methylation of histone H3K36, which activates the expression of IL-1β. Several substances, including lipopolysaccharide (LPS), IL-23, Epstein-Barr virus (EBV), and Pyridoxal-5'-phosphate (PLP), affect serine metabolism and join in the regulation of immunity. LPS can activate glycolysis and SSP processes. IL-23 promotes glycolysis and SSP processes through the activation of Mammalian Target of Rapamycin (mTOR) and hypoxia inducible factor-1α (HIF-1α), and activates SHMT2. Activating transcription factor 4 (ATF4) is also a downstream element of mTOR. It can activate the expression of enzymes involved in SSP and SHMT2. EBV up-regulates SHMT2 and MTHFD2 by targeting c-Myc. PLP can promote the transformation of SHMT2 from dimer to tetramer, keeping its catalytic function. While direct interaction with dimer-SHMT2 enhances the delivery of BRCC36 isopeptidase complex (BRISC) to ubiquitylated type I interferon (IFN) receptors, lead to the deubiquitylation of IFN receptors and limiting the endocytosis and lysosomal degradation of these receptors.

## Serine and antitumor immunity

Cell proliferation, such as that of cancer cells and immune cells, relies on nucleic acid and protein synthesis, which are influenced by the nutritional status of a cell, including its amino acid availability. As a pivotal NEAA, serine participates in multiple biosynthetic and signaling pathways. It can be converted into other kinds of amino acids or synthesized into important macromolecular substances such as phospholipids [39] and can also provide a carbon unit for one-carbon metabolism. Meanwhile, one-carbon metabolism takes part in the synthesis of purines and pyrimidines and produces cofactors such as NADPH, NADH and ATP [39-41]. In the end, serine is involved in the generation of S-adenosylmethionine (SAM), which is a ubiquitous methyl group donor, and the production of nicotinamide adenine dinucleotide phosphate (NADPH), which maintains cellular redox homeostasis. All these products are indispensable for cell proliferation, indicating that serine is irreplaceable in cells. It has been reported that rapidly proliferating effector T (Teff) cells in vitro and in vivo require serine supply [4]. Stimulated by tumor-specific antigens, primordial T cells transform into an active state characterized by rapid proliferation to generate a Teff cell pool that can mediate antitumor immunity. The activation of Teff cells requires the heavy consumption of various nutrient factors, including serine, to support their proliferative demands and exert effector functions. Likewise, dietary serine restriction can inhibit the expansion of T cells in the body [7]. Therefore, for tumor cells and activated immune cells, it is necessary to mobilize the pathways of serine acquisition to supply their demand, which is the theoretical basis for intervening in antitumor immunity from the perspective of serine metabolism (Fig. 2).

### The main metabolites derived from serine and antitumor immunity

Serine metabolism produces many metabolites, some of which play a pivotal role in antitumor immunity. First, NADPH is a coenzyme that acts as a hydrogen transporter in many biological reactions and can participate in various anabolic reactions as a reducing agent. NADPH has strong antioxidant capacity, which is beneficial for maintaining cell growth and prolonging lifespan [42]. A recent study indicated that Epstein-Barr virus (EBV) maintains NADPH levels in infected cells by upregulating import and synthesis of serine to augment one-carbon flux for B cell proliferation [43]. Mechanistically, EBV can increase the expression of methylenetetrahydrofolate dehydrogenase 2 (MTHFD2) by targeting MYC. MTHFD2 is a vital enzyme in one-carbon metabolism, and it can transform NADP^+^ to NADPH. In addition, T lymphocytes, as an important part of the adaptive immune system, have many biological functions, such as directly killing target cells and assisting B cells in producing antibodies and cytokines. Serine is considered a key immune metabolite that directly modulates adaptive immunity by controlling T cell proliferative capacity. Mechanistically, serine supplies one-carbon units for de novo nucleotide biosynthesis in proliferating T cells [7]. On the other hand, the influence of serine on the T cell immune response may rely on epigenetic effects. Serine can also initiate one-carbon metabolism to facilitate the generation of S-adenosylmethionine (SAM) in the methionine cycle. SAM is a direct methyl donor involved in the methylation of cellular DNA, RNA and proteins, and evidence suggests that lipopolysaccharide (LPS) activates the SSP and one-carbon metabolism, fueling SAM generation in macrophages [44]. SAM generation maintains a relatively high SAM:S-adenosyl-homocysteine (SAH) ratio to support the trimethylation of histone H3 lysine 36 (H3K36me3) for IL-1β production, and impairment of SAM generation pathways leads to anti-inflammatory outcomes, suggesting that SAM is an essential metabolite for inflammatory macrophages [44]. Therefore, serine is indispensable for the proliferation and differentiation of immune cells and the production of some immune cytokines mainly via NADPH-mediated antioxidant effects and SAM-induced methylation.

### The key regulators of serine metabolism and antitumor immunity

In the last section, we described the synthetic and metabolic pathways of serine, which are sensitive to a variety of regulatory factors and enzymes. Since serine is important in the proliferation and activation of immune cells, the mediators that affect serine metabolism may affect immune cells. Here, we describe several core pieces of evidence that connect serine metabolism to antitumor immunity. First, mammalian target of rapamycin (mTOR) is a serine-threonine kinase that coordinates nutrient and growth factor availability for cellular growth, division, and differentiation [45]. mTOR increases the synthesis and metabolism of serine by upregulating key enzymes [46] and plays important roles in both innate and adaptive immune responses [5]. In terms of T cells, mTOR can promote glycolysis and support effector T cell differentiation, growth and function [47]. For B cells, mTORC1 is highly activated during the pro- and pre-B stages of mouse B cell development [45]. Conditional disruption of the mTORC1 coactivating protein in developing mouse B cells leads to a developmental block at the pre-B cell stage [45]. In addition, mTOR inhibition treatments have been shown to be effective in B cell malignancies [48]. In innate immunity, multiple innate immune cells require mTOR-promoting serine metabolism to maintain growth and proliferation. For example, mTORC1 is stimulated in NK cells activated in vivo and in vitro, and mTORC1 activity is required for the production of the key NK cell effector molecule IFN-γ [49]. These processes require the upregulation of glucose uptake and glycolysis, and directly limiting the rate of glycolysis inhibits IFN-γ production [49]. In addition, IL-23 induces the activation of mTOR in neutrophils. Blockade of the mTOR pathway inhibits IL-23-induced IL-17 and IL-22 production [50]. The production of IL-17 and IL-22 also relies on the activation of HIF-1α induced by mTORC1 [51]. As a downstream element of mTOR, the upregulation of the transcription factor hypoxia inducible factor-1α (HIF-1α) also augments the expression of genes involved in intermediary metabolism and contributes to the rapid proliferation of Teff cells [47]. HIF-1α can increase glucose uptake and catabolism through glycolysis, supporting serine by supplying the necessary materials [48]. As another downstream element of mTORC1, activating transcription factor 4 (ATF4) also has an effect on T cells [52]. ATF4 is a key regulator of SSP, can directly activate the promoters of PHGDH, PSAT1 and SHMT2, upregulate their expression, and increase the synthesis and metabolism of serine [53]. ATF4-deficient CD4+ T cells have defects in redox homeostasis, proliferation, differentiation, and cytokine production [52]. This evidence suggests that the accelerated synthesis and metabolism of serine is beneficial for the cloning and amplification of T cells. In summary, the nutrient sensor mTOR has an important impact on serine synthesis and metabolism, and different immune cells need to complete their immune functions through this pathway, suggesting that altering mTOR may be regarded as a coupler linking serine metabolism and antitumor immunity.

SHMT2, which participates in serine and one-carbon metabolism, has recently been associated with the regulation of inflammatory cytokine signaling. In addition to the tetrameric form during metabolic activity, SHMT2 adopts a dimeric form without metabolic activity that can interact with BRCC36 isopeptidase complex (BRISC) deubiquitylase (DUB), thus influencing the type I interferon (IFN) response. Direct interaction with SHMT2 enhances the delivery of BRISC to ubiquitylated IFN receptors, allowing BRCC36 to deubiquitylate lysine-63-linked ubiquitin chains (Ub(K63)) on IFN receptors and limiting the endocytosis and lysosomal degradation of these receptors. In addition, pyridoxal-5'-phosphate (PLP), the active form of vitamin B6 and a cofactor of SHMT2, can promote the transformation of dimeric SHMT2 to tetrameric SHMT2. Increased intracellular PLP levels reduce the interaction between BRISC and SHMT2, accompanied by subdued inflammatory signaling [54]. Tetrameric SHMT2 can catalyze one-carbon unit metabolism, while the dimeric form of SHMT2 can enhance the IFN immune response, which seems to suggest that intracellular serine metabolism and immune flux are regulated by controlling exogenous vitamin B6. This evidence connects SHMT2—a metabolic enzyme—and the IFN response pathway, building a bridge between metabolism and immunity. These findings also provide insight into whether serine is involved in this process.

In the tumor microenvironment, the nutrition source of immune cells is insufficient, which weakens the antitumor immune response. Under conditions of glucose or glutamine deprivation, c-Myc, a crucial oncogene, can activate the SSP by upregulating the levels of key SSP enzymes (PHGDH, PSAT1 and PSPH) [31]. Similarly, the evidence shows that the upregulation of the transcription factor c-Myc supports the rapid proliferation of Teff cells [47]. Tumor cells also tend to reshape their serine metabolism pathways via mutations in oncogenes such as c-Myc to maintain their rapid proliferation [3, 55]. Thus, tumor cells and immune cells must compete for a limited amount of nutrients. Typically, immune cells lose, and without sufficient amino acids, glucose and other nutrients, immune cells cannot rapidly expand and induce an antitumor immune response, resulting in tumor immune escape and immunotherapeutic resistance. Therefore, serine is required in antitumor immunity, but supplementing serine is simply not enough. There are many questions that need to be answered for immune cells to take advantage of serine. Supportive immunity cannot rely only on serine supplementation, so we must determine how to help immune cells win the competition with tumor cells and utilize serine more effectively.

## Serine and senescence

**Figure 3.**
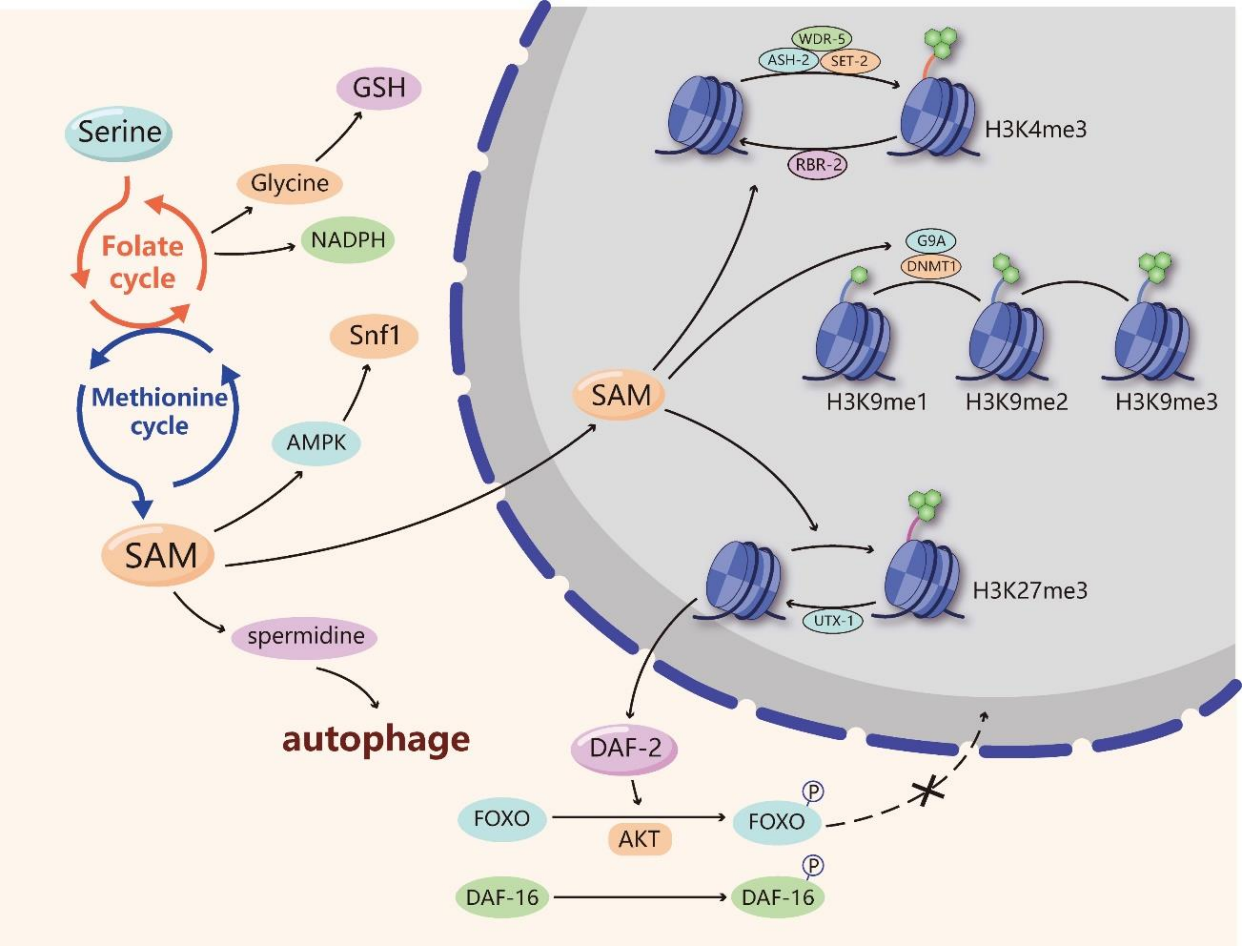
Serine and senescence. Serine produces various metabolites, such as NADPH, glutathione (GSH) and S-adenosylmethionine (SAM), by participating in one-carbon metabolism that includes the folate cycle and the methionine cycle. NADPH and GSH can hinder senescence by exerting antioxidant function. SAM enters the nucleus and is involved in the methylation of histone H3 lysine 4 (H3K4), histone 3 lysine 9 (H3K9) and histone H3 lysine 27 (H3K27). High levels of H3K4me3 adversely affect the longevity, while H3K9me1 and H3K27me3 are beneficial to longevity. Besides, the demethylase UTX-1 can remove the transcription repressive marker of H3K27me3 and increase the gene expression of the insulin/insulin-like growth factor (IGF)-1 receptor homolog DAF-2. DAF-2 induces the phosphorylation of FOXO/DAF-16 proteins through the AKT pathway, hindering their ability to enter the nucleus and activate anti-aging genes. SAM could also extend lifespan via activating the universal energy-sensing regulator Snf1 induced by AMP-activated protein kinase (AMPK). In addition, SAM can be involved in spermidine synthesis. Spermidine depends on functional autophagy to increase the autophagic turnover of cytoplasmic organelles or long-lived proteins.

Cellular senescence was originally identified as a process that permanently limits the proliferation of normal human cells in culture [56]. Recently, cell senescence has also been considered a stress response that can be induced by a variety of intracellular and extracellular factors [57], meaning that senescence can be considered a passive process caused by irreversible cell damage. Senescent cells change their metabolism and assist with the repair of normal tissues, which is a protective mechanism in the body. Meanwhile, amino acid dysfunction may contribute to the development of senescence, and the supply of some special amino acids may delay or promote senescence. Recently, it has been shown that serine can improve age-related disease. D-Serine is an agonist of N-methyl-D-aspartate receptors (NMDARs), which mainly function in synaptic communication. As aging progresses, endogenous levels of D-serine are reduced in the hippocampus, and supplementation with exogenous D-serine prevents age-related deficits of isolated NMDAR-dependent synaptic potentials [58]. Moreover, the D-serine-related pathway can be targeted by the age-related accumulation of ROS. Maintaining elevated D-serine levels in the aging hippocampus through the control of the redox state prevents injuries to the cellular mechanisms underlying cognitive aging [11]. Taken together, these results indicate an important connection between serine and senescence, and there seem to be several pathways through which serine participates in cell senescence (Fig. 3).

### Serine-mediated methylation and senescence

Serine metabolism can impact the intracellular methylated response through the production of SAM within the process of one-carbon metabolism. SAM is considered the major methyl donor during intracellular methylation reactions, including DNA/RNA and histone methylation [35]. Although serine is not directly involved in the methylation process, serine starvation reduces global levels of methylation in cancer cells by blocking the methionine cycle [59]. Meanwhile, several types of methylations, including DNA and histone methylations, can occur during the aging process [60, 61]. Therefore, we speculate that serine is involved in regulating senescence by affecting methylation. First, there is evidence that age-related methylation levels were different in longer-lived Ames dwarf mutated mice compared with normal mice. Further age-related methylation was suppressed in calorie-restricted and rapamycin-treated mice (rapamycin is an inhibitor of mTOR) and led to long longevity [62]. This evidence suggests that reduced age-related methylation levels contribute to longevity. Importantly, serine could influence longevity by regulating the methylation of various histone proteins and DNA. In terms of histone methylation, trimethylated histone H3 lysine 4 (H3K4me3) regulatory complexes are important for longevity. The lifespan of *Caenorhabditis elegans* (*C. elegans*) was found to be related to a complex consisting of ASH-2, WDR-5 and the H3K4me3 methyltransferase SET-2. The knockdown of ASH-2, WDR-5 or SET-2 in fertile worms significantly extended worm lifespan [63]. Worm SET2 was associated with the generation of H3K4me3 [64], and the knockdown or mutation of ASH-2, WDR-5 or SET-2 reduced global H3K4me3 levels in the larvae [63], indicating that ASH-2, WDR-5 and SET-2 affect lifespan by modulating H3K4me3 levels. In addition, RBR-2 is a homologous H3K4me3 demethylase whose absence led to an increase in H3K4me3 levels [65] and a decrease in the lifespan of wild-type worms, and the overexpression of RBR-2 prolonged the lifespan of *C. elegans* [63]. These studies suggest that high levels of H3K4me3 adversely affect the longevity of *C. elegans*. Since hypermethylated histones are generally considered inhibitory markers of gene transcription, H3K4me3 may accelerate aging by decreasing the transcription of genes that are beneficial to survival. The methylation of histone 3 lysine 9 (H3K9) also has an effect on longevity. The histone methyltransferase G9a can transfer methyl groups to H3K9, increasing the levels of dimethylated H3K9 (H3K9me2) [66]. During RAS-induced senescence, the degradation of G9a leads to a decrease in H3K9me2, reducing the expression of IL-6 and IL-8, which are important factors related to senescence [41]. In addition, the depletion of the methyltransferase Suv39h1 will reduce trimethylation of H3K9 (H3K9me3) levels, restore DNA repair capacity and delay senescence in a progeroid mouse model [55]. The evidence implies that monomethylation of H3K9 (H3K9me1) may be beneficial for a long lifespan, with H3K9me1 being associated with transcriptional activation and H3K9me2 with transcriptional repression [67]. In contrast, high levels of trimethylated histone H3 lysine 27 (H3K27me3) seem to be beneficial to worm longevity. H3K27me3 levels decreased significantly with age [68], and its demethylase UTX-1 mRNA levels increased with age in the worms [69]. Knockout of UTX-1 in worms resulted in increased H3K27me3 levels and extended the lifespan of the worms [69], which reflects the negative correlation between H3K27me3 and longevity. Mechanistically, UTX-1 can remove the transcription repressive marker of H3K27me3 and increase the gene expression of the insulin/insulin-like growth factor (IGF)-1 receptor homolog DAF-2. DAF-2 induces the phosphorylation of FOXO/DAF-16 proteins through the AKT pathway, hindering their ability to enter the nucleus and activate anti-aging genes, leading to decreased cell maintenance and cell function and senescence [69]. In terms of advanced creatures, it has also been found that a decrease in H3K27me3 can extend the lifespan of *Drosophila* [70]. A high level of aging-related H3K27me3 leads to a reduction in glycolysis gene expression. Polycomb repressive complexes (PRCs), which are important histone modification enzymes, can promote and maintain H3K27me3. The reduction in H3K27me3 due to PRC deficiency promotes glycolysis and a long lifespan [70]. Although H3K4, H3K9 and H3K27 have different effects on longevity, their methyl units are all provided by SAM, a metabolite of serine. The production of SAM can be regulated by controlling serine synthesis, which indirectly participates in the regulation of histone methylation and affects lifespan.

In addition to histone methylation, SAM can affect longevity by participating in DNA methylation, which plays a synergistic role with histone methylation in senescence. DNA methylation patterns are shaped by two opposing processes: adding and removing a methyl group at position five of cytosine in DNA [71]. DNA methylation is dynamic and depends on the activity of DNA methyltransferases (DNMTs) and dimethyl-transferases [72]. A recent study indicated that DNA methylation at some CpG islands tends to increase with age, while that at other CpG islands decreases [73, 74]. These age-related CpG methylation patterns have been combined into a tool for predicting age [74, 75]. CpG islands are unmethylated GC-rich regions with high densities of CpGs and are often correlated with promoter regions [71]. It is common for increased DNA methylation at a promoter to lead to decreased gene expression. However, this cannot be generalized, and not all age-related DNA methylation changes play a role in age-related diseases and symptoms. Global DNA hypomethylation is usually observed in age-related cancer. However, for some genes, such as insulin-like growth factor-II (IGFII), hypermethylated in cancer 1 (HIC1), caspase-8 (CASP8), glutathione S-transferase pi (GSTP1), suppressor of cytokine signaling 1 (SOCS1), RAS association domain family 1A (RASSF1A), p16/CDKN2A, adenomatosis polyposis coli (APC) and estrogen receptor 1 (ESR1), site-specific hyper-methylation of promoter regions is found in both aging and tumorigenesis [71]. In addition, some age-related neurodegenerative diseases, such as Alzheimer’s disease, show reduced levels of DNA methylation. The amyloid precursor protein (APP) gene of the Alzheimer’s brain exhibits gradual hypomethylation of the promoter [76]. Furthermore, DNA methyltransferase 1 (DNMT1) seems to be favorable for longevity. The level of DNMT1 is decreased in senescent cells. During DNA replication, maintenance of DNA methylation of the daughter strand is essential. DNA methyltransferase 1 (DNMT1) and the histone methyltransferase G9a coordinate the methylation of DNA and H3K9 at the replication site, maintaining the stable repression of genes during DNA replication [77]. Therefore, DNMT1 is required to keep cells proliferating and to resist aging. DNA methylation has an impact on cell senescence, but this impact is complicated. In general, some sites of DNA methylation increase with age and some decrease with age, and we can use these sites to predict aging. This also indicates that the role of serine in the effect of methylation regulation on senescence is complex. Additional studies are needed to determine whether other regulatory factors, such as methyltransferase and demethylase, are related to serine metabolism.

### Serine-related metabolic pathways and senescence

Serine can directly affect lifespan through metabolic regulation. As mentioned above, NADPH is an important metabolite of serine and participates in the senescence process. There is a negative relationship between NADPH and senescence. NADPH levels decrease with aging in several tissues, and the overexpression of NADPG-synthesizing enzymes gives rise to longer lifespans in some biological models [42]. In addition, serine can be converted into glycine by the catalysis of SHMT1/2 and then generate glutathione (GSH) [43]. GSH is synthesized from cysteine, glutamic acid and glycine. It is a vital antioxidant factor that helps maintain normal immune system function. GSH depletion has many deleterious effects, including impaired immune function and increased susceptibility to oxidants [78]. A decrease in GSH levels has been shown to be associated with aging and some pathological conditions, including neurodegenerative diseases [79]. Therefore, serine metabolism can maintain intracellular NADPH and GSH levels, and both hinder the cell senescence process. Moreover, SAM synthesis from serine can extend lifespan via activation of AMP-activated protein kinase (AMPK). Stimulating SAM synthesis activates the universal energy-sensing regulator Snf1, the *S. cerevisiae* ortholog of AMPK, resulting in lifespan extension [80]. SAM synthesis consumes both methionine and ATP. Methionine restriction extends lifespan and improves progeria in mice [81, 82], and metformin, the AMPK activator, retards aging in *C. elegans* by inducing methionine restriction [83]. Therefore, serine supplementation may promote SAM synthesis to prevent senescence. In addition, autophagy is a catabolic process through which macromolecules are degraded and recycled in the cell. Autophagy has been found to be increased in many long-lived model organisms and to contribute significantly to their longevity [84]. In contrast, the deletion of genes involved in autophagy shortens lifespan [85]. Serine supplementation increases the phosphorylation and S-glutathionylation of AMPKα [86], which further activates autophagy. Meanwhile, SAM can be converted into S-adenosyl-L-homocysteine (SAH), which further provides an aminopropyl group to synthesize spermidine [87]. Spermidine, a kind of natural polyamine, markedly extends the lifespan of yeast, flies, worms, and human immune cells [88-91]. Mechanistically, spermidine, a caloric restriction mimetic, induces protein deacetylation and depends on functional autophagy to increase the autophagic turnover of cytoplasmic organelles or long-lived proteins [92], which contributes to extending cell life.

In summary, serine can improve age-related diseases and may participate in the senescence process through several metabolites and metabolic pathways. From a metabolic perspective, serine supplementation can alleviate age-related diseases and aging symptoms. However, from the view of methylation regulation, the effect of serine on senescence is complicated. Both histone methylation and DNA methylation differ with age. Some methylation levels increase during aging, while others decrease. These discrepant phenomena indicate that there may be other substances involved in the regulatory pathways that assist serine in synergistically modifying senescent cell methylation. For methylation sites whose levels of methylation decrease with age, supplementation of SAM derived from serine can increase their methylation levels, thus delaying senescence. For methylation sites whose levels of methylation increase with age, attention should be paid to the inhibition of methylation at these sites. These findings may provide a new perspective on the importance of serine levels in aging regulation and an effective method for improving age-related diseases and alleviating aging symptoms.

## Conclusions and future perspectives

Various studies have shown that serine metabolism plays an integral role in antitumor immunity and senescence through diverse metabolic pathways and epigenetic regulation. Thus, serine and its downstream elements are promising targets for antitumor and anti-aging therapies. Although we already recognized that serine metabolism may be linked to immunity and senescence, little evidence and few macroscopic data sets are available, the details of which are unclear due to technological obstacles that prevent further research on serine metabolism. For example, the intracellular metabolic state is always in rapid transition and constant adjustment, and appropriate tissue extraction methods are essential for obtaining accurate snapshots of the metabolic state of cells and tissues in vivo, making correlation analysis extremely challenging. Moreover, it is far from sufficient to screen only a few histone and DNA methylation sites that participate in the epigenetic regulation of serine in senescent cells. It is more important to determine what other factors are involved in coordinating the methylation regulation of senescence-related sites.

The contents of nutrient substances, including serine, in the extracellular environment of tumor cells are usually insufficient. Notably, recent findings have revealed the influence of the tumor microenvironment on the immune response, achieved mainly through the utilization of intracellular and extracellular metabolites as signaling factors. We wonder whether tumor cells under these conditions secrete some signal factors to accelerate the synthesis of serine and the degradation of macromolecule proteins in surrounding tissues, and whether the obtained intracellular nutrients could be excreted into the tumor microenvironment and supplied to other tumor cells. If true, this hypothesis could explain why the capacity of T cells to acquire serine is inferior to that of tumor cells in the antitumor immune response. Because serine can be a neurotransmitter, we suspected that serine and its metabolites also play a role in the tumor microenvironment and antitumor immunity as a kind of signal transduction factor. These signaling factors might also have effects on the process of cell aging.

Aging itself is linked to cancer and immunity. The age-related accumulation of senescent cells has been shown to shorten both life span and health span in mice [93]. Among vertebrates, aging can promote hyperplastic pathologies, including cancer. In multicellular organisms with renewable tissues, aging means gain-of-function changes that allow cells to proliferate inappropriately, and these changes allow cells to increase their abilities to proliferate, migrate, and colonize ectopic sites; to survive hostile tissue environments; and to evade attack by the immune system, which are the hallmarks of cancer [94]. In addition, senescent cells acquire a senescence-associated secretory phenotype (SASP), which entails the chronic transcriptional induction and secretion of numerous proinflammatory cytokines, chemokines, growth factors and proteases. Although SASP can promote tissue repair and remodeling, it can also become maladaptive and promote aging phenotypes and pathologies when chronically present [93]. Aging also causes a decreased immune system. It has been reported that aging exerts different degrees of effects on various immune cells in both innate immunity and adaptive immunity, such as reduced migratory ability of neutrophils, delayed transcriptional response of peripheral blood mononuclear cells, and functionally deficient T cells [93]. Impaired immune function is related to declining health and a higher incidence of cancer in the elderly. Neutrophils and macrophages represent the first line of defense against tumor cells, yet their ability to phagocytose pathogens decreases with aging. Cytotoxic T lymphocytes are critical in eliminating tumors, but T cell function is also compromised with aging [95]. Thus, in aging tissues, antitumor immunity becomes more limited.

To exert a better antitumor immune response, two strategies can be taken into consideration. The first is to induce the senescence of tumor cells and break them into proliferating states. The other is to eliminate the senescence of immune cells and restore their normal immune function. The induction of senescence in cancer cells and the elimination of senescent cells by pharmacological interventions in aging tissues have been studied [96]. In this process, serine may be an intermediate bridge to combine senescence and antitumor immunity. As mentioned above, serine supplementation is beneficial to the growth, proliferation, and differentiation of immune cells and to anti-cell senescence. We hypothesize that serine can reduce tumor occurrence and enhance the antitumor response by exerting an anti-aging effect. For example, the redox state of the cell strongly influences T cell signaling. In T cells from aged individuals, ROS remain high, which would inhibit TCR signaling [93]. The metabolites of serine, NADPH and GSH both play antioxidant roles, so it is reasonable to speculate that serine can reverse the age-related ROS status of T cells by exerting antioxidant action to enhance the immune response of T cells. In addition, it is assumed that regular intake of nutrients involved in the metabolism of the methyl group, such as folic acid or vitamin B12, can slow down the gradual hypomethylation observed during the aging process [71]. Thus, supplementation of serine should also play a similar role fueling one-carbon metabolism and alleviating the age-related diseases associated with hypomethylation and tumor growth. That is, serine plays an important role in not only senescence and immunity but also in the connection of senescence and immunity. If the inner connections can be elucidated, the role of serine metabolism in cancer, immunity and senescence can inspire new therapeutic strategies and has the potential to alter the mode of cancer therapeutics and senescence intervention.
